# Supplementary material for: Speech sound discrimination in background noise across the lifespan: a comparative study in Mongolian gerbils and humans
Source: Front Aging Neurosci. 2025 Jun 9;17:1570305. doi: 10.3389/fnagi.2025.1570305 (PMC12183199; doi:10.3389/fnagi.2025.1570305)
Supplement: Supplementary file 1 [file Table_1.docx]

Supplementary Material


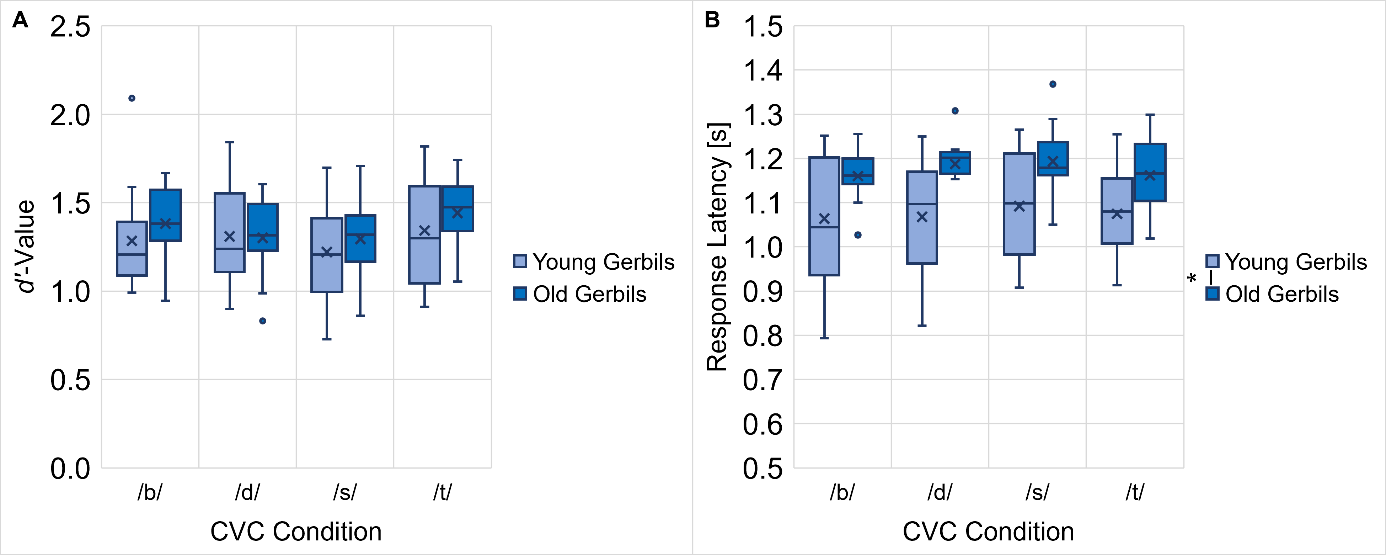


**Supplementary Figure 1.** Influence of different flanking consonants on vowel discrimination in gerbils. *: *p* < 0.05


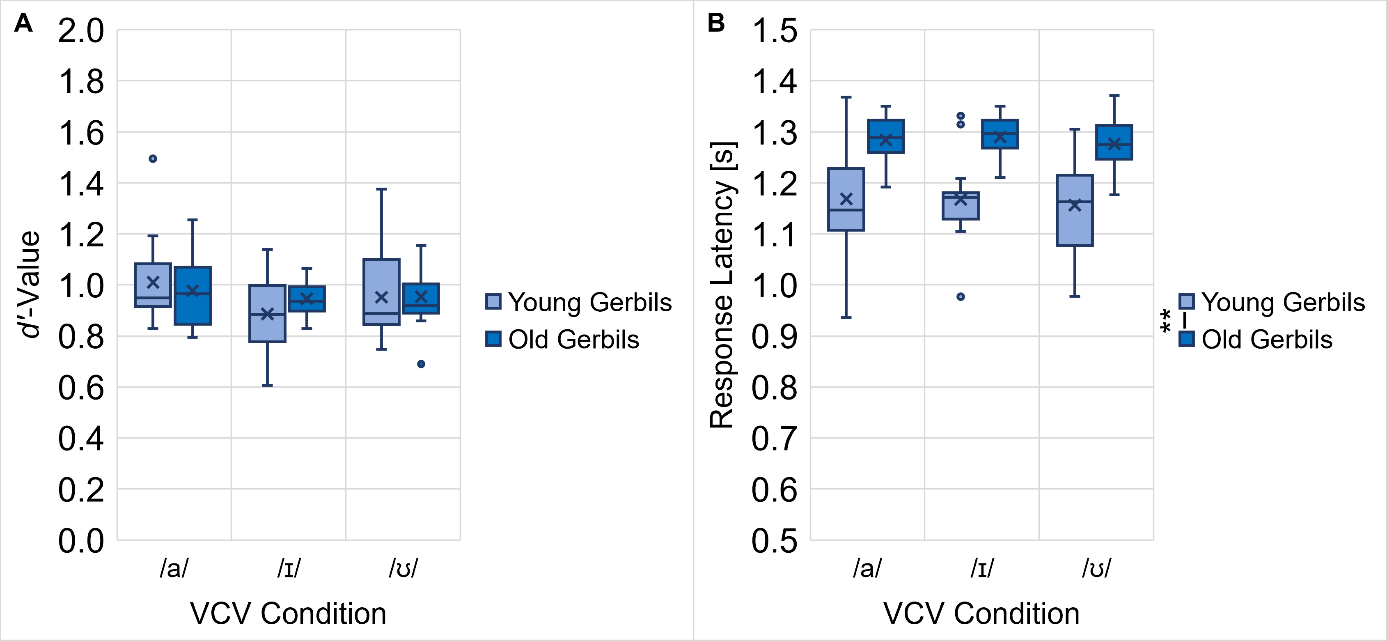


**Supplementary Figure 2.** Influence of different flanking vowels on consonant discrimination in gerbils. **: *p* < 0.01


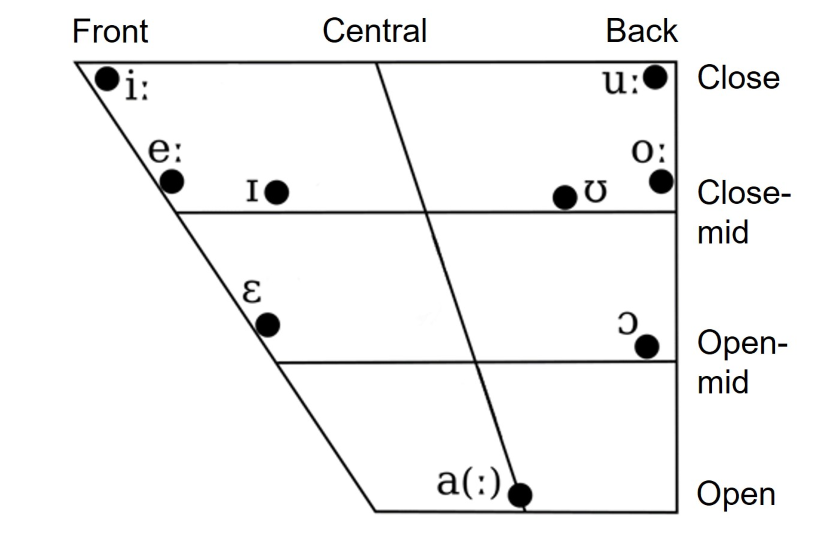


**Supplementary Figure 3.** Edited vowel chart for Northern Standard German [(Kleiner et al., 2015)](#_CTVL0015d9b80702b2f40c1bf7b44224eb2889c) showing the articulatory configurations of the vowels in the present study. The tongue backness ranges from front to back and the tongue height ranges from open to close.

**
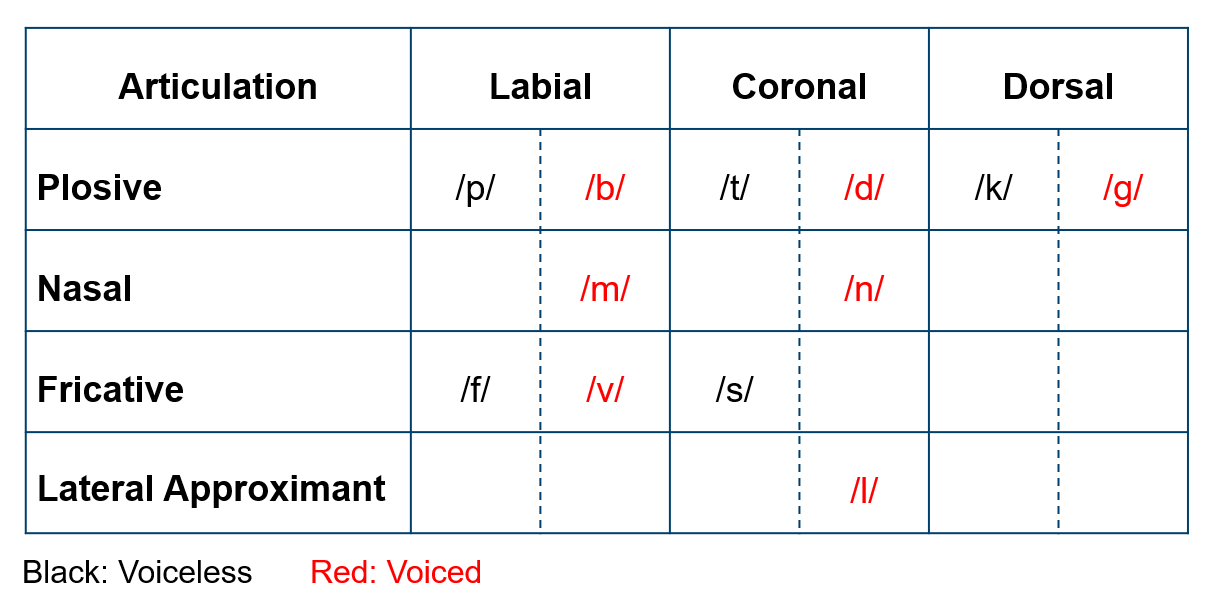
**

**Supplementary Figure 4.** Edited consonant chart [(International Phonetic Association, 2015)](#_CTVL001ef9d35b54edf423cbe454a1ef9a738db) showing the articulatory configurations of the consonants in the present study. The different rows and columns comprise different manners and places of articulation, respectively. The voicing characteristics can be differentiated by color.


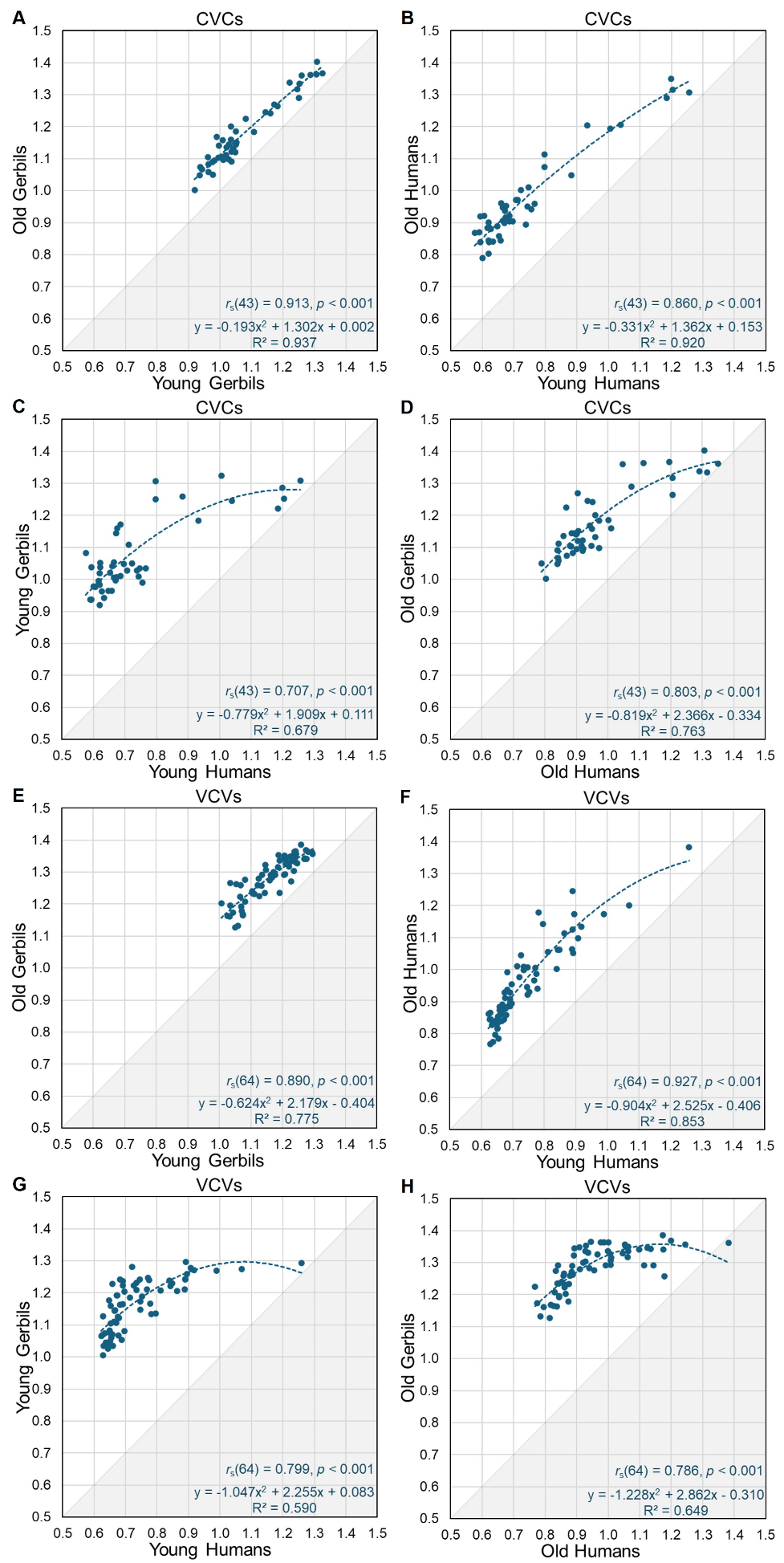


**Supplementary Figure 5.** Scatterplots with correlations between mean response latencies for different age groups of gerbils and humans for vowel and consonant discriminations.
